# Comparing consumer preferences for sustainable dairy activities among countries

**DOI:** 10.1007/s41237-022-00192-w

**Published:** 2023-01-20

**Authors:** Hideo Aizaki, Hironobu Takeshita

**Affiliations:** 1 Agricultural Economics, Research Faculty of Agriculture, Hokkaido University, Kita-9, Nishi-9, Kita-Ku, Sapporo, Hokkaido 060-8589 Japan; 2 Graduate School of Bioagricultural Sciences, Nagoya University, Furo-Cho, Chikusa-Ku, Nagoya, Aichi 464-8601 Japan

**Keywords:** Case 1 best–worst scaling, Consumer preference, Dairy farming, International comparison, Sustainability

## Abstract

This study measures consumer preferences for 11 sustainable dairy activities and examines the differences in preferences among five countries: the UK, the Netherlands, France, Italy, and Japan. A case 1 best–worst scaling is used to evaluate greenhouse gas emissions, fertilizer application, soil management, water management, biodiversity, working environment, animal care, wastes, market development, rural communities, and product safety and quality. Consumers across countries have diverse preferences for sustainable dairy farming activities, which may be related to the COVID-19 pandemic and social attention toward the environment and agriculture. Preferential differences for some activities were also revealed by gender and age. When discussing the priorities of some activities, conflicts between gender and generations could arise. Information on consumer preference can help various stakeholders discuss how to improve the sustainability of the dairy sector.

## Introduction

Progress toward the United Nations (UN) Sustainable Development Goals, which comprise 17 goals and balance among economic, social, and environmental sustainability, requires every sector in all countries to change their activities (UN 2015). As agriculture is an economic sector strongly linked to the environment, and agricultural products are essential for our daily lives, agricultural activities can be considered as important components in achieving these goals. Establishment of sustainable food and agricultural systems has been sought out globally (FAO 2021).

In 2016, the International Dairy Federation and the Food and Agriculture Organization of the United Nations signed the Dairy Declaration of Rotterdam, which stipulates sustainable development for the dairy sector following the UN 2030 Agenda for Sustainable Development (FAO 2016). Under the Declaration, the Dairy Sustainable Framework (DSF) formulated global criteria to promote activities that improve the economic, social, and environmental sustainability of the dairy sector. The 11 DSF criteria comprise greenhouse gas emissions, soil nutrients, soil quality and retention, water availability and quality, biodiversity, working conditions, animal care, wastes, market development, rural economies, and product safety and quality. Further, indicators were constructed to assess the degree of improvement for each criterion (DSF 2021).

The DSF criteria could enable cooperation among dairy sectors across countries to make their activities sustainable. However, it should be noted that international cooperation does not imply that all countries must adopt the same pathway to promote sustainable dairy sectors. A wide range of diverse dairy sectors exist around the world. Due to the economic, social, and environmental differences of the dairy sectors, the order of priority of the DSF criteria to promote sustainable activities may differ among countries. Understanding these differences and the background of the difference facilitates the mitigation of potential conflicts among countries regarding the progress of sustainable dairy systems internationally. The priority order of these criteria should, therefore, be compared internationally to advance the global and cooperative progress of sustainable activities across dairy sectors.

Discussions among stakeholders are essential to prioritize activities that improve the 11 criteria. Stakeholders include the suppliers of inputs for raw milk production; dairy farmers; and manufacturers, retailers, and consumers of milk and dairy products, among others. Thus, understanding stakeholders’ various priorities regarding the DSF criteria could promote an efficient discussion. Among these stakeholders, consumers play an important role because they are connected with dairy farming via market and non-market channels. In the market-based channel, milk and dairy products must satisfy consumer requirements to generate higher profits. Dairy farming variously impacts consumers without passing through the dairy product markets (i.e., non-market impacts or multifunctionality [Renting et al. 2009]); for example, dairy farming may contribute to the construction of beautiful rural landscapes that are positively perceived by consumers. However, it may also negatively impact consumers due to the reduction in water quality of rivers caused by livestock excrement.

This study measures consumer preferences for the DSF’s 11 sustainability criteria and examines the differences in preferences among the following five countries: the UK, the Netherlands, France, Italy, and Japan. These are major dairy farming countries in Europe and East Asia; however, their dairy sectors and consumer markets for milk and dairy products have economic, social, and environmental differences. Although the DSF criteria are defined for the entire dairy sector, this study focuses only on the production process for raw milk that involves dairy farmers, because consumers are relatively more interested in raw material production within the food chain (Jin and Zhou 2014; Aizaki and Sato 2020). Additionally, evaluating each process in the dairy sector according to the 11 criteria imposes a heavy psychological burden on respondents and may decrease the quality of responses: the number of objects to be evaluated is equal to the number of processes multiplied by the number of DSF criteria.

This paper overviews the previous consumer valuation studies on sustainable dairy activities and methodological issues related to measuring and comparing consumer preferences for activities among countries (Section 2); explains the design of an international survey and method for measuring consumer preferences (Section 3); compares consumer preferences for activities within a country, among countries, and among age and gender groups (Section 4); discusses the validity of the results compared to the related studies (Section 5); and provides suggestions for advancing stakeholder discussions on sustainable dairy farming, and presents limitations of the study (Section 6).

## Background

### Previous studies on consumer valuations of sustainable dairy activities

Consumer preferences for sustainability-related characteristics of dairy farming have been investigated by consumer valuation studies of dairy product characteristics, such as organic (Managi et al. 2008); carbon footprint (Canavari and Coderoni 2020); animal welfare (Napolitano et al. 2008); good agricultural practices (Aizaki et al. 2013); local (Wägeli et al. 2016); and so on (see e.g., Cecchini et al. 2018 for reviews of consumer valuations of sustainable food product characteristics). While these consumer valuation studies targeted a few sustainability-related characteristics of dairy farming, a comprehensive valuation is required to discuss how to promote sustainable dairy farming.

Few consumer valuation studies have examined broadly sustainable dairy farming conditions. Nicholas et al. (2014) and Mandolesi et al. (2015) interviewed three types of stakeholders in the dairy supply chain (33 farmers, 30 retailers/processors, and 36 consumers) from four European countries to collect information regarding their valuations of 34 innovations that increase the sustainability of organic and low-input dairy supply chain systems. Ellison et al. (2017) elicited 264 US consumer preferences for seven practices related to four dairy and livestock products. Further, Jackson et al. (2020) investigated 2054 UK citizen preferences for 13 attributes of cow management, while Schiano et al. (2020) gauged 608 US consumer preferences for 27 dairy product attributes related to sustainability.

The previous studies provide valuable information that can be used for designing and managing stakeholders’ discussions on dairy sustainability. However, these studies have limitations: the sample sizes are limited (Nicholas et al. 2014; Mandolesi et al. 2015); the samples are gathered from single countries (Ellison et al. 2017; Jackson et al. 2020; Schiano et al. 2020); and the targeted practices are unbalanced from the perspective of three aspects—economic, social, and environmental—of sustainability (Ellison et al. 2017). Constraints related to the samples reduce the generalizability of the results and the unbalanced practices cannot be disregarded while studying the three aspects of dairy farming sustainability. Therefore, there is a lack of large sample-size study that compares consumer preferences for dairy farming conditions among countries to advance international stakeholders’ discussions on sustainable dairy farming practices.

The present study attempts to fill the gap by targeting consumers in five European and East Asian countries and focusing on 11 sustainable dairy activities based on the DSF criteria that reflects the three aspects of sustainability.

### Age and gender effects on consumer valuation of sustainable dairy activities

Based on social and psychological studies on consumer/citizen perceptions of environment/sustainability (Gifford and Nilsson 2014; Barone et al. 2020; Sánchez-Bravo et al. 2021), various consumer characteristics could affect their preferences for sustainable dairy activities. However, this study focuses only on the effects of age and gender, because consumer representatives among stakeholders discussing sustainable dairy farming should be selected according to population demographics—primarily defined by age and gender (Sénit et al. 2017).

The previous related studies have found that consumers’ age and/or gender are statistically significant factors that affect their understanding of sustainability concepts (Barone et al. 2020; Sánchez-Bravo et al. 2021) and their preferences for food values and agricultural and food policies (Bazzani et al. 2018; Caputo and Lusk 2020; Abe et al. 2021; Cerroni et al. 2022). However, this study did not hypothesize the effects of age and gender on consumer valuation for dairy sustainable activities and designed an explanatory analysis.

### Methods for measuring and comparing preferences among countries

For an international comparison of consumer preferences, this study uses case 1 (object case) best–worst scaling (BWS1). BWS1 is a stated preference method used to efficiently gauge people’s preferences for many items (Finn and Louviere 1992; Louviere et al. 2015). A list of items is established for evaluation, and respondents are asked to select the best and worst items from a subset of items. This style of questioning is repeated as items are changed in the subset. The statistical analysis of the responses reveals the relative preferences for the items.

Questions based on rating scales, which have been applied widely in studies on consumer valuation of food and food characteristics, may have limitations in the case of international comparison studies. Respondents’ social and cultural factors may produce biased responses: people in one country may be more likely to select a specific response, such as a midpoint scale “neutral.” Additionally, as respondents rate items without considering trade-offs between items in general style rating scale questions, they may give the same response (e.g., extremely agree) to items even when their true priority for an item differs from that of other items.

BWS1 can tackle these limitations. BWS1 asks respondents to select “the best” and “the worst” items from a set of items; due to this (i), the respondents cannot select the same response for the items and (ii) the discrimination issue of rating scale format can be avoided. Thus, BWS1 has been used in international comparison studies across various research fields (e.g., Auger et al. 2007; Cohen 2009; Lee et al. 2011; Cheung et al. 2018). In the field of food research, BWS1 studies have compared consumer food values between the USA and Norway (Bazzani et al. 2018), Japan, China, and Korea (Jo and Lee 2021), and Japan, Taiwan, and Indonesia (Yang et al. 2021), as well as investigated the factors that affect wine purchasing among several countries (Cohen 2009). For details on the advantages of using BWS1 for the international comparison of preferences for items, refer to Cohen (2003, 2009), Muller Loose and Lockshin (2013), and Heo et al. (2022).

Previous related studies on consumer food valuations frequently used the discrete choice experiment (DCE) (e.g., Aizaki 2012; Lizin et al. 2022 for reviews of DCEs in food research). The DCE, which is also known as choice-based conjoint analysis, is a stated preference method, which expresses an alternative (profile) as a bundle of attribute levels (characteristics of goods/services). It asks respondents to select their most preferred profile from a set of profiles, and reveals preferences for attribute levels by conducting a discrete choice model analysis of the responses. For example, Aizaki et al. (2013) measured consumer preferences for milk that comprised the following three attributes: good agricultural practice (GAP) certification label; hazard analysis and critical control points certification label; and price. The DCE approach is advantageous because it facilitates the measurement of consumer preferences regarding non-monetary characteristics of food products based on monetary units when using price as a product characteristic (e.g., consumers’ willingness to pay for the GAP label). A limitation of the approach is that the number of characteristics used as profile attributes for DCEs is restricted to manage respondents’ psychological burden, which increases as the number of attributes increases (Caussade et al. 2005). Furthermore, when comparing the results among countries, currency values among the countries must be exchanged appropriately (e.g., Aoki et al. 2017). As consumer preferences for the 11 criteria are not necessarily converted into monetary units to examine how to promote an effective discussion among stakeholders, we concluded that using BWS1 is more appropriate than DCEs for this study. To the best of our knowledge, the present study is the first to apply BWS1 and compare consumer valuation of sustainable dairy farming conditions among countries in Europe and East Asia.

## Materials and methods

### Web survey design

An international Web survey was conducted between January and February 2021. For each country, 1030 respondents were recruited from an online panel provided by a Web survey company, MACROMILL. Respondents were limited to those who usually purchased food products and daily necessities for their families and lived in major areas such as the capital and its surrounding areas.^1^ The proportions of respondents for each country were restricted by gender and age to examine the effects of these variables on respondents’ preferences. For each country, half of the respondents were male and 206 respondents were placed in each of the following age categories: 20s, 30s, 40s, 50s, and 60s. The proportion of respondents by gender and age may differ from those in the consumer population for a given country. This sampling approach, which recruits an equal number of respondents across respondent categories, has been used in consumer valuation studies (e.g., Hartmann et al. 2016; Aizaki and Sato 2020; Michel et al. 2021).

Questionnaires were prepared in British English, Dutch, French, Italian, and Japanese. A Japanese questionnaire was drafted and then translated into English. The expressions and terms between the Japanese and English editions were adjusted with support from an interpreter who specialized in agriculture. Subsequently, the Dutch, French, and Italian editions were translated from the English edition. The accuracy of these translations was improved by informing the translators about the adjustments between the Japanese and English editions.

### Scenario and choice sets design for BWS1

As previously mentioned, the DSF criteria were designed to measure the degree of sustainability of activities spanning the dairy sector (DSF 2021). However, this study focuses only on the activities of dairy farmers involved in the production process of raw milk. Therefore, the DSF criteria were modified as follows (terms in the parentheses are short descriptions used in each BWS1 question; those in square brackets are abbreviations).^2^

- Quantifying emissions of greenhouse gases and working on their reduction (Working on reducing greenhouse gas emissions) [GHG].
- When applying fertilizers, working on minimizing ﻿impact on water and air quality while maintaining/improving soil quality (Working on applying fertilizers while taking care of the water and air) [FERTILIZER].
- Actively working on the conservation of soil quality to ensure that optimum productivity is obtained (Working on soil management suited to production) [SOIL].
- Managing the amount of use and drainage of water to ensure that quantitative and qualitative impacts on local rivers and groundwater are minimized (Caring about the amount of water use and drainage management) [WATER].
- Working on understanding positive and negative, direct and indirect impacts on biodiversity and maintaining/enhancing biodiversity (Caring about biodiversity) [BIODIVERSITY].
- Building an environment in which workers can work safely, and respecting/promoting their rights (Caring about the working environment of workers) [WORKER].
- Treating dairy cattle with care so that they are free from hunger and thirst, discomfort, pain, injury and disease, fear and distress, and are able to engage in relatively normal patterns of animal behavior (Caring about the physical and mental health of dairy cattle) [CATTLE].
- Working to reduce the amount of wastes as much as possible and to reuse and/or recycle wastes (Working on reducing wastes). Do not include animal feces in wastes [WASTE].
- Trying for a new business with use of dairy farming in collaboration with milk/dairy processors and distributors (Trying for a new business with use of dairy farming) [BUSINESS].
- Contributing to maintaining/improving the resilience and economic viability of rural communities (Contributing to maintaining/improving rural communities) [COMMUNITY].
- Safeguarding the integrity and transparency of the business, so as to ensure the optimal nutrition, quality and safety of raw milk (Working on ensuring the quality and safety of raw milk) [MILK].

The BWS1 questions assume a situation wherein subsidies funded by taxpayers are used to support dairy farmers in the respondents’ countries. They also assume that subsidies are not given uniformly to all dairy farmers, who can be prioritized based on their farming activities, which correspond to the 11 DSF criteria.

**Fig. 1:**
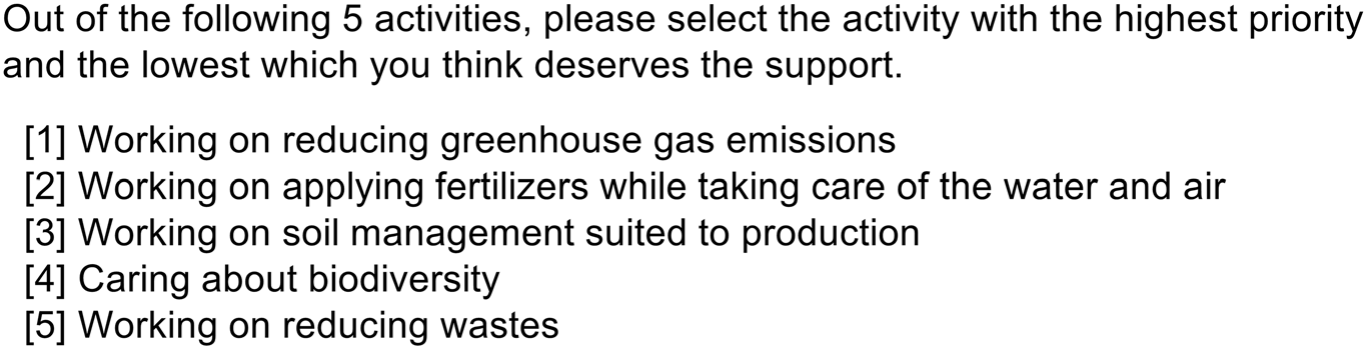
Example of a best–worst scaling question. As the survey Web site interactively processes responses to the questions, an activity selected as the best disappears when asking respondents to select the worst activity in each question

After explaining the situation and criteria, respondents were asked to answer the BWS1 questions. A balanced incomplete block design (BIBD) with 11 treatments, 5 treatments per block, and 11 blocks (Cochran and Cox 1957) was used to construct the BWS1 questions. The 11 treatments of the BIBD are distinguished using 11 integer values from 1 to 11. The 11 integer values correspond to the 11 activities in the order mentioned above (i.e., GHG = 1, …, MILK = 11), and a row of the BIBD represents a combination of treatments that correspond to activities presented in a BWS1 question. For example, one row of the BIBD comprises five integer values: 1, 2, 3, 5, and 8. This means that the BWS1 question corresponding to that BIBD row represents the following five activities: GHG, FERTILIZER, SOIL, BIODIVERSITY, and WASTE (Fig. 1). The respondents were asked to select the best and worst activities. This style of question was repeated 11 times because there were 11 BWS1 questions per respondent, which corresponded to the number of blocks of the BIBD. Using the functionalities of the Web survey system, an activity selected as “best” disappeared when respondents were asked to select the worst activity for each question. Further, the orders of the 11 BWS1 questions and five activities per question were randomized.

Compared with rating scale questions, BWS1 questions are easy for respondents to answer, but some respondents might not appropriately consider the activities displayed in each BWS1 question. This study did not exclude such respondents from the valid sample, as the survey did not include question that measured the respondents’ efforts during the BWS1 questions.

### Methods for measuring preferences

Responses to BWS1 questions are analyzed based on the following two approaches: count analysis and modeling analysis (Louviere et al. 2015; Aizaki and Fogarty 2021). The count analysis measures consumer preferences for items using scores calculated from the number of times each item is selected as the best and the worst in the BWS1 survey, whereas the modeling analysis uses coefficients estimated by fitting a discrete choice model to the BWS1 survey data to achieve the same. The modeling approach is based on the random utility theory and, thus, is suitable for investigating theoretically the fundamental behavior of respondents’ choices in BWS1 questions. However, the main objective of this study is to measure consumer preferences for the 11 criteria and compare those among countries. Furthermore, Flynn et al. (2007, 2008) and Louviere et al. (2015) highlighted that the results of the count analysis are on average similar to those of the modeling analysis. For reference, the correlation coefficients among count-based scores for the 11 activities, which are defined below, and the share of preferences (Cohen 2003; Lusk and Briggeman 2009) for the 11 activities, which are calculated from estimates of conditional logit maximum difference, marginal, and marginal sequential model analyses (Flynn et al. 2007, 2008; Hensher et al. 2015; Louviere et al. 2015) of the data set in this study (these are omitted), are larger than 0.99. Therefore, this study employed the count-based approach, which can be implemented with ease.

There are two major variants of count-based-scores: the best-minus-worst score and the ratio of best and worst scores. For respondent *n*, the best-minus-worst score of item *i* is defined as ${B}_{in}-{W}_{in}$, while the ratio of best and worst scores is defined as $\sqrt{{B}_{in}/{W}_{in}}$, where ${B}_{in}$ and ${W}_{in}$ are the number of times respondent *n* selected item *i* as the best and worst in all the BWS1 questions, respectively. For example, respondent *n* selected item *i* twice as the best and once as the worst in the 11 BWS1 questions, resulting in ${B}_{in}=2$ and ${W}_{in}=1$; ${B}_{in}-{W}_{in}=1$; and $\sqrt{{B}_{in}/{W}_{in}}=\sqrt{2}$. This study used an aggregated version of the standardized best–worst ratio score (BW score), which is calculated as:

$$\frac{\sqrt{{B}_{i}/{W}_{i}}}{{\sum }_{j=1}^{11}\sqrt{{B}_{j}/{W}_{j}}},$$

where ${B}_{i}$ is the sum of the best scores for item *i* across all respondents (${B}_{i}={\sum }_{n=1}^{N}{B}_{in}$); and ${W}_{i}$ is the sum of the worst scores for item *i* across all respondents (${W}_{i}={\sum }_{n=1}^{N}{W}_{in}$). Compared with the best-minus-worst score, the ratio score is suitable for international comparison studies because the best-minus-worst score only reflects the difference in scores among activities, while the ratio score reveals the relative importance of items. Additionally, dividing the ratio score for item *i* by that for item *j* indicates how much item *i* is preferred to item *j*. These features enable the easy interpretation of the results even when the reference (i.e., the most or least important) item differs among countries (Cohen 2009; Mueller Loose and Lockshin 2013). Compared with the disaggregated variant of the ratio score, it is highly probable that the aggregated variant can avoid division by zero as the aggregated worst score ${W}_{i}$ seldom takes the value of 0, which applies to this study as well.

This study implements four comparisons of BW scores: (i) a comparison of BW scores for activity *i* and *j* by country; (ii) a comparison of BW scores for activity *i* between any two countries; (iii) a comparison of BW scores for activity *i* between males and females by country; and (iv) a comparison of BW scores for activity *i* among any two age categories by country. A statistical test of the differences in BW scores between any two groups is divided into the following steps: (i) 30,000 bootstrap samples for each group are generated from the original data set; (ii) the empirical distributions of BW scores, for items by group, are constructed from the bootstrap samples; and (iii) the Poe test (Poe et al. 1997, 2005) is implemented to examine whether the distribution of the BW scores in a group differs statistically from that in another group. The Poe test for the first case is implemented as a comparison of dependent distributions (Poe et al. 1997), while those for the remaining three are implemented as independent distributions (Poe et al. 2005). When comparing BW scores by country, activities with insignificant differences are grouped into the same rank group. The significance level (*α*) was set at 0.05 (5%). Further, a Bonferroni correction is applied since multiple Poe tests are conducted simultaneously.

R (R Core Team 2022) and the add-in packages support.BWS (Aizaki 2021; Aizaki and Fogarty 2023; Aizaki et al. 2014), mded (Aizaki 2015; Aizaki et al. 2014), and survival (Therneau 2022; Therneau and Grambsch 2000) are used to prepare a dataset for analysis, calculate BW scores, and conduct the statistical analysis.

## Results

### Comparing scores by country

Table 1 presents the ranks of the 11 activities based on BW scores by country. Column “Rank” indicates the order of activities by country, which follows point estimates of the scores shown in column “Score.” Column “Group” indicates rank groups of activities with insignificant differences in scores. The first priority activity differs among the five countries, while BUSINESS emerged at the bottom of the priority list in all the countries. The relative importance of BUSINESS ranges from 3.1% (0.031) in Italy to 5.2% (0.052) in the Netherlands. In the UK, the Netherlands, and Italy, GHG takes the first priority and is approximately 4.3 (= 0.150/0.035), 2.8, and 4.7 times higher, respectively, than BUSINESS. The highest priority activity is BIODIVERSITY in France and MILK in Japan, which are approximately 3.7 and 3.4 times higher, respectively, than BUSINESS.

**Table 1:** Scores and ranks for activities by country

Country and activity	Score	Rank	Group							
			1	2	3	4	5	6	7	8
UK										
GHG	0.150	1	1							
MILK	0.110	2		2						
CATTLE	0.107	3		2						
WASTE	0.107	4		2						
WORKER	0.100	5		2	3					
WATER	0.091	6			3	4				
FERTILIZER	0.087	7			3	4				
BIODIVERSITY	0.083	8				4				
COMMUNITY	0.067	9					5			
SOIL	0.064	10					5			
BUSINESS	0.035	11						6		
Netherlands										
GHG	0.148	1	1							
CATTLE	0.127	2	1	2						
WASTE	0.113	3		2	3					
FERTILIZER	0.100	4			3	4				
WATER	0.089	5				4	5			
MILK	0.086	6				4	5			
BIODIVERSITY	0.078	7					5	6		
WORKER	0.071	8						6	7	
SOIL	0.070	9						6	7	
COMMUNITY	0.066	10							7	
BUSINESS	0.052	11								8
France										
BIODIVERSITY	0.139	1	1							
GHG	0.124	2	1							
WASTE	0.104	3		2						
CATTLE	0.100	4		2	3					
FERTILIZER	0.096	5		2	3	4				
WATER	0.093	6		2	3	4				
WORKER	0.088	7			3	4				
MILK	0.084	8				4				
SOIL	0.073	9					5			
COMMUNITY	0.062	10						6		
BUSINESS	0.038	11							7	
Italy										
GHG	0.148	1	1							
FERTILIZER	0.142	2	1							
WASTE	0.112	3		2						
WATER	0.105	4		2						
CATTLE	0.103	5		2						
WORKER	0.099	6		2						
BIODIVERSITY	0.072	7			3					
MILK	0.069	8			3					
SOIL	0.069	9			3					
COMMUNITY	0.050	10				4				
BUSINESS	0.031	11					5			
Japan										
MILK	0.154	1	1							
WORKER	0.119	2		2						
GHG	0.111	3		2	3					
WASTE	0.109	4		2	3					
FERTILIZER	0.097	5			3	4				
COMMUNITY	0.094	6			3	4				
WATER	0.089	7				4				
SOIL	0.075	8					5			
CATTLE	0.055	9						6		
BIODIVERSITY	0.051	10						6	7	
BUSINESS	0.045	11							7	

As the distribution of the BW scores for GHG in the UK differs significantly from those for the remaining ten activities, the first priority group comprises only GHG (the first column in the “Group” columns). MILK is ranked in the second priority group; however, the Poe test results indicate that MILK differs insignificantly from CATTLE, WASTE, and WORKER. As such, the second priority group comprises these four activities. Since an activity can be grouped into one or more statistical test-based groups, several activities are common in two or more groups. Conversely, the fifth priority group comprises COMMUNITY and SOIL, which do not belong to other groups. The sixth priority group is also an isolated group comprising only BUSINESS. Similarly, the 11 activities are divided into five groups in Italy, seven groups in France and Japan, and eight groups in the Netherlands.

### Comparing scores among countries

Table 2 indicates the Poe test results comparing the distribution of BW scores for activities among countries. The numbers of significant differences vary across activities: there is no significant difference for WASTE between any pair of countries, while there are significant differences for MILK between nine pairs of countries. With an increased number of significant differences for an activity, consumer valuations of the activity among countries are more diverse in terms of the BW score. However, a linear relationship does not necessarily exist between the number of significant differences (Table 2) and ranks of activities based on the point estimates and statistical tests (Table 1). Thus, the Poe tests among activities and ranks of activities are integrated, resulting in the 11 activities being classified into three groups: a stable group in which activities have relatively stable scores and ranks among countries; an unstable group in which activities have relatively unstable scores and ranks among countries; and a middle group which comprises the remaining activities.

**Table 2:** Comparison of scores and ranks for activities among countries

Activity	UK		Netherlands		France		Italy		Japan	
GHG	0.150^a^	(1) [1]	0.148^a^	(1) [1]	0.124^b^	(2) [1]	0.148^a^	(1) [1]	0.111^c^	(3) [2, 3]
FERTILIZER	0.087^c^	(7) [3, 4]	0.100^b^	(4) [3, 4]	0.096^bc^	(5) [2, 3, 4]	0.142^a^	(2) [1]	0.097^bc^	(5) [3, 4]
SOIL	0.064^b^	(10) [5]	0.070^ab^	(9) [6, 7]	0.073^a^	(9) [5]	0.069^ab^	(9) [3]	0.075^a^	(8) [5]
WATER	0.091^b^	(6) [3, 4]	0.089^b^	(5) [4, 5]	0.093^b^	(6) [2, 3, 4]	0.105^a^	(4) [2]	0.089^b^	(7) [4]
BIODIVERSITY	0.083^b^	(8) [4]	0.078^bc^	(7) [5, 6]	0.139^a^	(1) [1]	0.072^c^	(7) [3]	0.051^d^	(10) [6, 7]
WORKER	0.100^b^	(5) [2, 3]	0.071^d^	(8) [6, 7]	0.088^c^	(7) [3, 4]	0.099^bc^	(6) [2]	0.119^a^	(2) [2]
CATTLE	0.107^b^	(3) [2]	0.127^a^	(2) [1, 2]	0.100^b^	(4) [2, 3]	0.103^b^	(5) [2]	0.055^c^	(9) [6]
WASTE	0.107^a^	(4) [2]	0.113^a^	(3) [2, 3]	0.104^a^	(3) [2]	0.112^a^	(3) [2]	0.109^a^	(4) [2, 3]
BUSINESS	0.035^bc^	(11) [6]	0.052^a^	(11) [8]	0.038^b^	(11) [7]	0.031^c^	(11) [5]	0.045^a^	(11) [7]
COMMUNITY	0.067^b^	(9) [5]	0.066^b^	(10) [7]	0.062^b^	(10) [6]	0.050^c^	(10) [4]	0.094^a^	(6) [3, 4]
MILK	0.110^b^	(2) [2]	0.086^c^	(6) [4, 5]	0.084^c^	(8) [4]	0.069^d^	(8) [3]	0.154^a^	(1) [1]

Same superscripts in each row indicate insignificant differences in score distribution. Values in parentheses and brackets are “Rank” and “Group” in Table 1, respectively

The stable group comprises WASTE, SOIL, BUSINESS, and GHG. The distributions of BW scores for WASTE differ insignificantly between all pairs of countries, while the rank order of WASTE is relatively high in all countries. It has the third or fourth place in the point estimate-based ranking, and the second to third priority groups in the statistical test-based ranking. The distributions of BW scores for SOIL differ significantly between the UK and France and between the UK and Japan. SOIL ranks relatively low in all countries, that is, the eighth to tenth priority (the third to seventh priority groups). Although the distributions of BW scores for BUSINESS differ significantly among seven out of the ten pairs of countries, it occupies the lowest priority rank in all the countries. The distributions of BW scores for GHG differ among seven pairs of countries. However, GHG occupies the first priority group in four countries, and belongs to the second and third priority groups in Japan.

There are five unstable activities among the countries: WORKER, MILK, BIODIVERSITY, CATTLE, and COMMUNITY. The score distributions differ significantly for WORKER in eight out of ten pairs of countries. Ranks of WORKER vary largely from the second place in Japan to the eighth place in the Netherlands, which correspond to the second to seventh priority groups. The difference in the score distribution for MILK is significant among nine out of ten pairs of countries. MILK is in the first and second priority (group) in Japan and the UK, respectively, while it ranks the sixth (the fourth and fifth groups) in the Netherlands and the eighth (the third and fourth groups) in France and Italy. Among eight out of ten pairs of countries, there are significant differences in score distribution for BIODIVERSITY. The ranks of BIODIVERSITY among the five countries vary widely, from the first priority in France to the tenth priority (the sixth and seventh groups) in Japan. The score distributions for CATTLE differ significantly in seven out of ten pairs of countries. CATTLE is ranked relatively high (the second to fifth priority) in four European countries, while it is ranked ninth in Japan. Rank groups of CATTLE also vary from the first to the sixth. The distributions of BW scores for COMMUNITY differ significantly in seven pairs of countries. COMMUNITY is ranked as the sixth priority in Japan and the ninth or tenth priority in other countries (the fourth to seventh priority groups).

The remaining two activities, WATER and FERTILIZER, belong to the middle group, which represent four and five significant differences out of ten pairs of countries, respectively. WATER ranks from the fourth to seventh priority (the second to fifth groups) and FERTILIZER from the second to seventh priority (the first to fourth groups).

### Comparing scores among gender and age categories by country

Table 3 compares BW scores and ranks for activities between the male and female groups by country. Four activities have a significant difference in score distribution between males and females in the UK, three in Italy and Japan, and two in the Netherlands and France. Significant differences in score distribution by gender were observed in all the countries for BUSINESS and four for CATTLE. Among the five countries, the direction of gender effects on scores is the same for CATTLE and BUSINESS: scores for CATTLE among males are less than those among females; and scores for BUSINESS among males are greater than those among females. However, differences in the ranks of CATTLE and BUSINESS between gender groups exist among the countries. The difference in the ranking of BUSINESS between males and females is narrow in the five countries: one in Japan (the tenth in males and the eleventh in females) and zero (the same rank) in other cases. The difference in the ranking of CATTLE between males and females varies among the five countries: one in the Netherlands (the third in males and the second in females), two in the UK (the fourth in males and the second in females) and Japan (the eleventh in males and the ninth in females), and three in France and Italy (the sixth in males and the third in females). In the UK, difference in score distribution for WORKER is significant between males and females, and the difference in ranking is somewhat wide (the sixth in males and the third in females).

**Table 3:** Scores and ranks for activities between gender categories by country

Country and activity	Score		Rank	
	Male	Female	Male	Female
UK				
GHG	0.141	0.159	1	1
FERTILIZER	0.091	0.083	7	7
SOIL^*^	0.075	0.054	9	10
WATER	0.092	0.089	5	6
BIODIVERSITY	0.090	0.075	8	8
WORKER^*^	0.091	0.111	6	3
CATTLE^*^	0.096	0.117	4	2
WASTE	0.103	0.110	3	4
BUSINESS^*^	0.040	0.029	11	11
COMMUNITY	0.070	0.063	10	9
MILK	0.112	0.108	2	5
Netherlands				
GHG	0.143	0.153	1	1
FERTILIZER	0.098	0.100	4	4
SOIL	0.073	0.068	8	9
WATER	0.095	0.084	5	6
BIODIVERSITY	0.079	0.077	7	7
WORKER	0.069	0.072	9	8
CATTLE^*^	0.111	0.143	3	2
WASTE	0.117	0.109	2	3
BUSINESS^*^	0.059	0.046	11	11
COMMUNITY	0.068	0.064	10	10
MILK	0.088	0.084	6	5
France				
GHG	0.120	0.129	2	2
FERTILIZER	0.096	0.097	5	5
SOIL	0.077	0.068	9	9
WATER	0.097	0.090	4	7
BIODIVERSITY	0.147	0.130	1	1
WORKER	0.084	0.091	8	6
CATTLE^*^	0.091	0.109	6	3
WASTE	0.099	0.108	3	4
BUSINESS^*^	0.042	0.034	11	11
COMMUNITY	0.062	0.062	10	10
MILK	0.085	0.083	7	8
Italy				
GHG	0.150	0.146	1	2
FERTILIZER	0.133	0.151	2	1
SOIL	0.071	0.067	9	8
WATER	0.112	0.098	3	6
BIODIVERSITY	0.075	0.067	8	7
WORKER	0.095	0.101	5	5
CATTLE^*^	0.088	0.120	6	3
WASTE	0.110	0.115	4	4
BUSINESS^*^	0.037	0.025	11	11
COMMUNITY	0.053	0.047	10	10
MILK^*^	0.076	0.063	7	9
Japan				
GHG	0.104	0.119	4	3
FERTILIZER	0.104	0.089	3	6
SOIL^*^	0.082	0.067	8	8
WATER	0.094	0.084	6	7
BIODIVERSITY^*^	0.059	0.044	9	10
WORKER	0.113	0.125	2	2
CATTLE	0.052	0.058	11	9
WASTE	0.103	0.115	5	4
BUSINESS^*^	0.055	0.037	10	11
COMMUNITY	0.091	0.096	7	5
MILK	0.142	0.165	1	1

An activity with an asterisk indicates a significant difference in score between male and female

Table 4 compares BW scores and ranks for activities among the five age categories by country. The numbers of significant differences in score distributions between age category pairs are limited: 11 cases in the UK; five cases in the Netherlands; nine cases in France; one case in Italy; and four cases in Japan. Differences in the ranks corresponding to significant cases in Italy and the Netherlands are two or less, while larger differences in ranks are observed in some cases, in which significant differences are found. Four and five differences in ranks correspond to the three cases in Japan: people in their 30s and 50s rank GHG as the sixth and the second, respectively; those in their 20s and 60s rank WASTE as the second and the sixth, respectively; and those in their 30s and 40s rank COMMUNITY as the third and the eighth, respectively. Similarly, maximum differences in ranks, which correspond to the significant cases, are five (WASTE) and seven (MILK) in the UK, and ﻿four (WASTE) and five (WATER and MILK) in France.

**Table 4:** Scores and ranks for activities by age category and country

Country and activity	Score					Rank				
	20s	30s	40s	50s	60s	20s	30s	40s	50s	60s
UK										
GHG	0.139^a^	0.137^a^	0.146^a^	0.165^a^	0.156^a^	1	1	1	1	2
FERTILIZER	0.080^a^	0.098^a^	0.100^a^	0.073^a^	0.086^a^	8	5	6	8	6
SOIL	0.063^a^	0.063^a^	0.072^a^	0.061^a^	0.060^a^	10	10	9	9	10
WATER	0.094^a^	0.105^a^	0.083^a^	0.082^a^	0.088^a^	5	4	7	7	5
BIODIVERSITY	0.090^a^	0.082^a^	0.073^a^	0.099^a^	0.069^a^	7	7	8	4	8
WORKER	0.097^a^	0.108^a^	0.106^a^	0.095^a^	0.091^a^	4	3	4	6	4
CATTLE	0.100^a^	0.095^a^	0.103^a^	0.116^a^	0.116^a^	3	6	5	3	3
WASTE	0.115^a^	0.128^a^	0.111^ab^	0.095^ab^	0.083^b^	2	2	2	5	7
BUSINESS	0.054^a^	0.040^ab^	0.030^bc^	0.023^c^	0.026^bc^	11	11	11	11	11
COMMUNITY	0.076^a^	0.068^a^	0.068^a^	0.060^a^	0.061^a^	9	9	10	10	9
MILK	0.093^bc^	0.076^c^	0.107^b^	0.131^ab^	0.165^a^	6	8	3	2	1
Netherlands										
GHG	0.155^ab^	0.121^b^	0.139^ab^	0.147^ab^	0.180^a^	1	1	2	1	1
FERTILIZER	0.092^a^	0.098^a^	0.108^a^	0.098^a^	0.099^a^	5	5	4	4	4
SOIL	0.075^a^	0.075^a^	0.069^a^	0.066^a^	0.062^a^	9	8	8	8	9
WATER	0.096^a^	0.097^a^	0.083^a^	0.086^a^	0.080^a^	4	6	6	5	6
BIODIVERSITY	0.077 ^a^	0.082^a^	0.087^a^	0.078^a^	0.066^a^	8	7	5	7	7
WORKER	0.081^a^	0.075^a^	0.067^a^	0.064^a^	0.065^a^	7	9	9	9	8
CATTLE	0.101^b^	0.110^ab^	0.140^a^	0.143^a^	0.153^a^	3	2	1	2	2
WASTE	0.110^a^	0.105^a^	0.118^a^	0.128^a^	0.105^a^	2	3	3	3	3
BUSINESS	0.058^ab^	0.064^a^	0.044^b^	0.046^ab^	0.049^ab^	11	11	11	11	11
COMMUNITY	0.072^a^	0.073^a^	0.063^a^	0.062^a^	0.060^a^	10	10	10	10	10
MILK	0.083^a^	0.100^a^	0.081^a^	0.082^a^	0.081^a^	6	4	7	6	5
France										
GHG	0.138^a^	0.117^a^	0.123^a^	0.113^a^	0.130^a^	2	2	2	3	2
FERTILIZER	0.078^b^	0.089^ab^	0.096^ab^	0.100^ab^	0.117^a^	6	6	6	4	3
SOIL	0.071^a^	0.076^a^	0.079^a^	0.071^a^	0.066^a^	8	9	8	9	9
WATER	0.076^c^	0.086^bc^	0.104^ab^	0.115^a^	0.090^abc^	7	7	3	2	5
BIODIVERSITY	0.139^a^	0.127^a^	0.132^a^	0.139^a^	0.157^a^	1	1	1	1	1
WORKER	0.100^a^	0.095^a^	0.079^a^	0.083^a^	0.080^a^	5	5	9	8	8
CATTLE	0.102^a^	0.109^a^	0.102^a^	0.098^a^	0.087^a^	4	3	5	6	6
WASTE	0.122^a^	0.109^ab^	0.102^ab^	0.100^ab^	0.085^b^	3	4	4	5	7
BUSINESS	0.044^ab^	0.049^a^	0.037^abc^	0.033^bc^	0.026^c^	11	11	11	11	11
COMMUNITY	0.060^a^	0.067^a^	0.059^a^	0.061^a^	0.062^a^	10	10	10	10	10
MILK	0.070^b^	0.077^ab^	0.087^ab^	0.087^ab^	0.101^a^	9	8	7	7	4
Italy										
GHG	0.178^a^	0.135^a^	0.136 ^a^	0.150^a^	0.147^a^	1	1	1	2	2
FERTILIZER	0.126^b^	0.131^ab^	0.131^ab^	0.173^a^	0.152^ab^	2	2	2	1	1
SOIL	0.069^a^	0.065^a^	0.076^a^	0.069^a^	0.064^a^	8	9	7	8	9
WATER	0.110^a^	0.118^a^	0.111^a^	0.100^a^	0.088^a^	4	4	4	4	6
BIODIVERSITY	0.076^a^	0.070^a^	0.075^a^	0.066^a^	0.069^a^	7	7	9	9	8
WORKER	0.090^a^	0.094^a^	0.095^a^	0.092^a^	0.119^a^	6	6	6	6	3
CATTLE	0.104^a^	0.107^a^	0.098^a^	0.097^a^	0.109^a^	5	5	5	5	4
WASTE	0.111^a^	0.124^a^	0.111^a^	0.112^a^	0.104^a^	3	3	3	3	5
BUSINESS	0.036^a^	0.035^a^	0.032^a^	0.024^a^	0.029^a^	11	11	11	11	11
COMMUNITY	0.049^a^	0.052^a^	0.059^a^	0.042^a^	0.047^a^	10	10	10	10	10
MILK	0.053^a^	0.069^a^	0.076^a^	0.076^a^	0.072^a^	9	8	8	7	7
Japan										
GHG	0.116^ab^	0.084^b^	0.118^ab^	0.125^a^	0.114^ab^	4	6	2	2	2
FERTILIZER	0.089^a^	0.091^a^	0.105^a^	0.105^a^	0.093^a^	6	5	5	5	4
SOIL	0.065^a^	0.078^a^	0.077^a^	0.072^a^	0.081^a^	8	8	7	8	8
WATER	0.085^a^	0.082^a^	0.100^a^	0.093^a^	0.084^a^	7	7	6	6	7
BIODIVERSITY	0.054^a^	0.058^a^	0.056^a^	0.040^a^	0.050^a^	10	9	9	10	10
WORKER	0.135^a^	0.131^a^	0.110^a^	0.107^a^	0.112^a^	1	2	4	4	3
CATTLE	0.055^a^	0.057^a^	0.056^a^	0.049^a^	0.059^a^	9	10	10	9	9
WASTE	0.131^a^	0.105^ab^	0.111^ab^	0.109^ab^	0.089^b^	2	4	3	3	6
BUSINESS	0.043^a^	0.052^a^	0.046^a^	0.039^a^	0.044^a^	11	11	11	11	11
COMMUNITY	0.099^ab^	0.115^a^	0.077^b^	0.092^ab^	0.091^ab^	5	3	8	7	5
MILK	0.127^b^	0.147^ab^	0.145^ab^	0.169^ab^	0.183^a^	3	1	1	1	1

Same superscripts in each row indicate insignificant differences in BW ratio score distribution

## Discussion

This section discusses the validity of the results compared with the related studies and surveys. Interestingly, activities to reduce greenhouse gas emissions and for new business with dairy farming have stable ranks among the five countries. Reduction of greenhouse gas emissions occupies the first priority (group) in the four European countries, while it ranks lower in Japan. The highest evaluation of greenhouse gas emissions in European countries is similar to the Eurobarometer results, which revealed that a reduction in greenhouse gas emissions occupied the first and second highest priorities among serious global problems in 2021 (EU 2021a) and 2019 (EU 2019b), respectively. Another survey revealed that Japanese citizens indicate that the greenhouse gas emissions issue is the most important environmental issue that Japan faces, but environmental issue ranks third among social issues in Japan (Murata 2021). The Japanese sense of crisis regarding greenhouse gas emissions may be slightly lower than that of citizens in European countries, which may be reflected in its lower evaluation in Japan compared to the other four countries. Activity for new business ranks the lowest in the priority list among all the countries; however, the reason behind this occurrence remains ambiguous. As an ill-founded conjecture, consumers might be skeptical of striking a balance among the economic, social, and environmental aspects of sustainability, or they might consider sustainability to have a narrow meaning: their understanding of the concept of sustainability might lack somewhat of an economic perspective (see, e.g., Sánchez-Bravo et al. 2021 for diversity in citizens’ perceptions of the sustainability concept).

The BWS1 analysis found that the ranks of activities that support the working environment for workers, ensure the quality and safety of raw milk, promote biodiversity, consider the physical and mental health of dairy cattle, and contribute to maintaining/improving rural communities differ among countries. Supporting the working environment ranks higher in Japan than in the other four countries. However, previous food value studies have revealed that Japanese consumers rank fairness, which partly includes a value corresponding to the activity for workers in this study, relatively lower (Jo and Lee 2021; Abe et al. 2021). Although the definition of fairness in previous food value studies is broader than that of the activity for workers in this study, a significant factor causing the discrepancy may be a high awareness among the Japanese regarding the working environment because of the COVID-19 pandemic and corresponding limitations on daily activities (e.g., staying at home), which has social impacts (e.g., decrease in income and increase in unemployment). For example, US consumer preferences for fairness increased significantly during the COVID-19 pandemic compared with before the pandemic (Cerroni et al. 2022).

Ensuring the quality and safety of raw milk occupies the utmost priority in Japan, while it ranks lower in the other four countries. In previous studies on food valuation, the said activity corresponds to safety, taste, and nutrition. Food value studies in the USA and Norway (Bazzani et al. 2018) and in Japan (Jo and Lee 2021; Abe et al. 2021) have revealed that safety was the first to third most important value, while taste and nutrition were the second to fifth most important values. Compared with these studies, the activity to ensure the quality and safety of milk ranks relatively lower in France, the Netherlands, and Italy. Consumers in the three countries might perceive the additional support for dairy farmers’ activities related to the quality and safety of raw milk as less important because they satisfy the quality and safety standards of milk and dairy products of their countries.

The rank of promoting biodiversity among the five countries varies widely, from the first priority group in France to the sixth and seventh group in Japan. Biodiversity occupies the utmost priority in France, which is supported by the results of the Eurobarometer survey on attitudes toward biodiversity (EU 2019a). The survey revealed that 71% of French respondents, which is the highest percentage among 28 EU countries, believe that intensive farming, intensive forestry, and over-fishing threaten biodiversity substantially. This awareness is consistent with the highest priority attributed to dairy farmers’ activity regarding biodiversity in this study by French respondents. Conversely, the Japanese Government conducted an opinion poll on the environment, which included a question on the knowledge regarding the term “biodiversity”; 20% of the respondents answered, “I know what it means,” 32% answered, “I have heard of it but I do not know what it means,” and 47% answered, “I have never heard of it” (COJ 2019). This result suggests that Japanese citizens are less familiar with the term biodiversity than European citizens, of whom 41% know what it means; 30% have heard of it but do not know what it means; and 29% have never heard of it (EU 2019a). Accordingly, the activity for biodiversity may rank the lowest in Japan among the five countries.

Activity for caring about the physical and mental health of dairy cattle ranks higher in the four European countries but lower in Japan. This activity corresponds to animal welfare in previous studies on food value. Animal welfare ranked lower in Japan (Jo and Lee 2021; Abe et al. 2021), while it ranked middle in the USA and Norway (Bazzani et al. 2018) and higher in the UK (Jackson et al. 2020). A contemporary concept of animal welfare was developed in western countries and subsequently introduced in Japan. Thus, it may be less well known in Japan than in the remaining four countries (Sasaki et al. 2022).

Contributing to maintaining/improving rural communities is ranked relatively higher in Japan, while it is ranked lower in the remaining countries. The relatively higher rank in Japan might be attributed to Japanese awareness of the relationship between agriculture and rural communities: historically, agriculture has been connected to the local community although the relationship has weakened over time. Agricultural water is managed by farmer organizations formed in rural communities. Farmer organizations that provide agricultural services are established and managed locally. Furthermore, some traditional activities, such as festivals, are related to agriculture (e.g., praying for a good harvest). Although these activities and organizations are not necessarily related to dairy farming, the relationship between agriculture and rural communities has been widely recognized. For example, a recent opinion poll (COJ 2021) indicated that 86.5% of respondents believe that rural areas play an important role in food production, which is the highest among the seven roles explicitly mentioned.

Two activities, caring about the amount of water use and drainage management and working on applying fertilizers while taking care of the water and air, belong to the middle group, for which four and five significant differences exist out of ten pairs of countries, respectively. The relatively higher rank of the activity regarding fertilizer application in Italy might be related to organic farming. The share of the organic area in the total utilized agricultural area in Italy is 15%, which is the highest among the five countries considered in this study (EU 2021b; MAFFJ 2020). Organic farming must follow the rules pertaining to the usage of chemicals, including fertilizer. Therefore, Italian consumers’ awareness of fertilizer application in agriculture might be higher than that in the other countries.

For the effects of age and gender, there are three remarkable results: females attached more importance to the activity for the physical and mental health of dairy cattle than males in countries except for Japan (note that the rank of the activity for cattle differs between males and females even in Japan); consumers in their 20s gave higher scores to the activity for reducing wastes than those in their 60s in the UK, France, and Japan; and consumers in their 60s gave higher scores to ensuring the quality and safety of raw milk than those in their 20s in the UK, France, and Japan. Among these results, the effect of gender on the activity for cattle health and that of age on the quality and safety of raw milk seem to be supported by previous related findings: females pay more attention to animal welfare than males (McKendree et al. 2014); and older people may pay more attention to the quality and safety of milk than younger people because they are more concerned about their health (MHLWJ 2020). The effect of age on the activity for reducing wastes is somewhat surprising as it highlights a completely different perspective than that found in previous studies: people’s attitudes toward and behavior regarding waste management are positively correlated with their age. For example, older people tend to generate less food wastes than younger people (Secondi et al. 2015). However, one study also suggested that younger people are more likely to support sustainability than older people (Yamane and Kaneko 2021). Thus, further investigation of the effect of age on reducing wastes is needed. Additionally, we could not discern why the second and third findings were not obtained in the Netherlands and Italy.

## Conclusion

Case 1 best–worst scaling was used to investigate consumer preferences for 11 activities of sustainable dairy farming among the following five countries: the UK, the Netherlands, France, Italy, and Japan. In each country, the 11 activities were ranked on the basis of measured preferences (scores) for activities. Reducing greenhouse gas emissions emerged as the highest priority activity in the UK, the Netherlands, and Italy; to promote biodiversity in France; and to ensure the quality and safety of raw milk in Japan, while new businesses with dairy farming had the lowest ranking in all five countries. The 11 activities were also divided into five to eight groups following the statistical test of difference in scores. The highest and lowest priority activities did not belong to two or more groups. This suggests that conflicts could arise among consumers while discussing the priority levels of activities, except for the highest and lowest priority activities.

This study also revealed that activities are classified into three groups on the basis of comparing scores and ranks of activities among the five countries. First, consumer valuations of activities to reduce waste, for soil management suited to production, for new businesses with dairy farming, and to reduce greenhouse gas emissions are stable among the countries. Second, activities that support the working environment, ensure the quality and safety of raw milk, promote biodiversity, consider the physical and mental health of dairy cattle, and contribute to maintaining/improving rural communities are unstable. Third, the valuations of activities that manage water use and drainage and apply fertilizers while preventing water and air pollution are moderate. The unstable consumer preferences in countries suggest that sustainable dairy farming activities should be promoted according to the economic, social, and environmental situation of the dairy sector in each country. This implies that experiences in the implementation of activities could differ among countries. Thus, sharing diverse experiences with each other could contribute toward accelerating the progress of sustainable dairy activities internationally.

Preferential differences were revealed by gender and age. The difference in the rank of caring about the physical and mental health of dairy cattle between males and females varies among the countries from one (the Netherlands) to three (France and Italy). Some age groups revealed relatively large differences in the ranking of working on reducing greenhouse gas emissions (Japan), working on reducing wastes (the UK, France,﻿ and Japan), contributing to maintaining/improving rural communities (Japan), working on ensuring the quality and safety of raw milk (the UK and France), and caring about the amount of water use and drainage management (France). Prioritizing activities with large differences according to gender and age could cause conflicts among consumers. Therefore, we should ensure that the ages and gender of consumers participating in stakeholder discussions on the sustainability of the dairy sector are fully representative of the population. When the number of consumer representatives has to be limited, information on preferences for activities among age and gender categories can be used to re-classify consumers into smaller groups based on the similarities in their preferences.

This study has some limitations. First, the sample is obtained from a Web survey, and the ratios of males to females and age categories are restricted. As such, the results of this study may be biased. Second, except for age and gender, other factors affecting differences in preferences for activities were not considered. For stakeholder discussions to progress efficiently, we should investigate the relationship between consumer preference for activities and other factors such as their knowledge of the dairy sector and the concept of “sustainability.” Third, all the respondents were treated as valid without considering how they effectively responded to the BWS1 questions. This might reduce the reliability of the results. Finally, since establishing a sustainable dairy sector is a global issue, additional comparison studies should include countries in other continents and developing countries that produce raw milk, as well as other stakeholders such as dairy farmers, manufacturers, and retailers of milk and dairy products.

## Data Availability

We have no permission to share the dataset as it was provided by the Japan Dairy Association.

## Footnotes

1 The survey area included the following: for the UK, City of London, City of Westminster, Kensington and Chelsea, Hammersmith and Fulham, Wandsworth, Lambeth, Southwark, Tower Hamlets, Hackney, Islington, Camden, Brent, Ealing, Hounslow, Richmond, Kingston, Merton, Sutton, Croydon, Bromley, Lewisham, Greenwich, Bexley, Havering, Barking and Dagenham, Redbridge, Newham, Waltham Forest, Haringey, Enfield, Barnet, Harrow, and Hillingdon; for the Netherlands, Amsterdam, Rotterdam, Den Haag, Utrecht, Eindhoven, and Tilburg; for France, Paris, Department of Seine-et-Marne, Department of Yvelines, Department of Essonne, Department of Hauts-de-Seine, Department of Seine-Saint-Denis, Department of Val-de-Marne, and Department of Val-d’Oise; for Italy, Province of Roma, Province of Milano, Province of Torino, Province of Firenze, and Province of Bologna; and for Japan, Tokyo Metropolis, Chiba Prefecture, Saitama Prefecture, and Kanagawa Prefecture.

2 Although the English edition of the questionnaire was written in British English, US English is used below.
